# Effect of Acute Stress Glycemic Control and Long-Term Glycemic Control on the Incidence of Post-Operative Infection in Diabetics Undergoing Cardiac Surgery

**DOI:** 10.7759/cureus.14031

**Published:** 2021-03-22

**Authors:** Sean R Bennett, Yazed M Alayesh, Aliah M Algarni, Ohoud D Alotaibi, Abdullah A Aladnani, Jose Andres Fernandez, Miriam R Bennett

**Affiliations:** 1 Anesthesiology, King Faisal Cardiac Center, King Abdullah Medical City, Jeddah, SAU; 2 College of Medicine, King Saud Bin Abdulaziz University for Health Sciences, Jeddah, SAU; 3 Academic Medicine, Manchester University Foundation Trust, Manchester, GBR

**Keywords:** diabetes, cardiac surgery, post-operative infection, stress glycemia

## Abstract

Objective

Post-operative infection after cardiac surgery causes prolonged hospital stay and increased mortality. In patients with diabetes, peri-operative and pre-operative glycemic control have been associated with increased risk of post-operative infection. Saudi Arabia is the 7th highest country in the world for the prevalence of diabetes. In our surgical population the incidence of diabetes is 77%. We were aware of a high incidence of post-operative infections in our institution. The aim of this work was to assess how peri-operative and pre-operative glycemic control was related to the six-week incidence of post-operative infection.

Method

We retrospectively collected data for 174 adult patients with diabetes undergoing cardiac surgery between January 2017 and June 2019. For group analysis of peri-operative glycemic control, a mean value of ≤10 mmol/l was categorized as optimal control and a mean value of >10 mmol/l as sub-optimal control. The admission glucose value, the maximum glucose value and glycosylated hemoglobin A1c (HbA1c) were separately recorded. Admission HbA1c was used for optimal long-term control group (HbA1c ≤ 7%) and sub-optimal long-term control group (HbA1c > 7%).

Results

Of the 174 patients 60 (34%) developed infection in the six-week post-operative period. No statistically significant difference in infections was seen in the optimal peri-operative control group (n = 24, 14%) compared to sub-optimal peri-operative control group (n = 36, 21%; p = 0.113). However, patients with infection had a significantly higher mean glucose (10.4 mmol/l versus 9.9 mmol/l, p = 0.0316) than no infection.

Grouping according to their HbA1c: well controlled group (41, 24.0%) and poor control group (130, 76.0%) showed no difference in infections. However, patients with lower HbA1c had better glycemic control as measured by: initial glucose (r = 0.52, p=<0.001); mean peri-operative glucose (r = 0.45, p=<0.001); maximum recorded glucose (r = 0.41, p=<0.001).

Conclusion

The majority of our patients presented with sub-optimal long-term glycemic control which we linked to poor stress glycemic control perioperatively. Patients with post-operative infections had higher mean peri-operative blood glucose. With the high incidence of diabetes in Saudi Arabia we have demonstrated the importance of good pre-operative assessment which allows tighter peri-operative glycemic control to reduce post-operative morbidity.

## Introduction

Post-operative infection after major cardiac surgery is a serious concern for the cardiothoracic team and the patients for whom they care. Worldwide the prevalence of post-operative infection is between 5 and 21% with prolonged hospital stays and higher mortality [1]. The most common post-operative infections are respiratory, surgical site and urinary tract infections. Pre-existing diabetes has been associated with increased risk of post-operative infection [2-4]. Poor control of underlying diabetes, measured as a raised glycosylated hemoglobin A1c (HbA1c) mmol/mol, also increases the risk of infection in some analysis but not all [5,6]. Efforts to better control the peri-operative glucose levels have been shown to decrease the incidence of post-operative infections [3].

According to the World Health Organization, Saudi Arabia is the 7th highest country in the world for the prevalence of diabetes at up to 50% in its adult population [7]. Our own data has shown that in our surgical patients the incidence of diabetes is 77% [8].

The aim of this work was to assess if peri-operative glycemic control was related to the six-week incidence of post-operative infection in a group of diabetic adult patients undergoing non-emergency cardiothoracic surgery in Saudi Arabia. Secondary objectives were to determine if pre-operative HbA1c effects the post-operative infection rate and whether pre-operative HbA1c impacts on the ability to establish optimal perioperative glycemic control.

## Materials and methods

The study was approved by the Institutional Review Board (reference JED-19-427780-109065) of King Abdullah International Medical Research Center.

The data was extracted from the inpatient medical records for all the adult diabetic cardiac surgical patients at the King Faisal Cardiac Center from January 2017 to June 2019. According to our blood glucose management protocol the target blood glucose was 6-10 mmol. Treatment was by intravenous insulin until the patient started an oral diet at which point, they returned to their pre-operative medication. All blood glucose values reported from the day of surgery and for 48 hours after surgery were collated to give a mean value of peri-operative glycemic control. For group analysis of peri-operative glycemic control, a mean value of ≤10 mmol/l was categorized as optimal control and a mean value of >10 mmol/l as sub-optimal control [5]. The admission glucose value, the maximum glucose value and HbA1c were also separately recorded. The admission HbA1c value was used to analyze optimal long-term control group (HbA1c ≤ 7%) and sub-optimal long-term control group (HbA1c >7%).

Statistics

Data analysis was done using SAS statistical package, version 9.4 (SAS Institute Inc., Cary, NC) and also on SPSS version 23 (IBM Corp., Armonk, NY). We used a univariant analysis approach, with a p-value <0.05 considered as significant. Data was analyzed in a non-continuous and continuous way as the standard deviations were similar. For comparisons between categorical data Chi-square test was used. For correlations between continuous data Spearman coefficient was assessed for non-parametric data and Pearson coefficient for parametric data.

## Results

Patient characteristics and complete blood glucose data were collected for 174 patients who underwent cardiac surgery. Eighty-four (48%) patients were classified as having optimal peri-operative glycemic control (mean glucose ≤10 mmol/l) and 90 (52%) were classified as sub-optimal (mean glucose >10 mmol/l). These glycemic control groups are shown in Table 1. The groups were evenly matched with no statistically significant difference between the two groups for BMI, gender and mortality.

**Table 1:** Peri-operative blood glucose control groups Patient characteristics, post-operative infection and Peri-operative Blood Glucose values according to optimal control (mean blood glucose ≤10 mmol/l) and sub-optimal control (mean blood glucose >10 mmol/l) groups. BMI = Body Mass Index (%), SEM = Standard Error of the Mean, *p < 0.05.

	All Patients (n = 174)	Optimal Control mean glucose <10 mmol (n = 84)	Sub-optimal Control mean glucose >10 mmol (n = 90)	p-value
Age years, mean (SEM)	62.4 (9.6)	62.3 (9.7)	62.5 (9.5)	0.902
BMI kg/m^2 ^mean (SEM)	29.6 (4.6)	29.1 (4.3)	30.1 (4.3)	0.175
Gender				0.943
Male (%)	128 (73.6)	62 (73.8)	66 (73.3)	
Female (%)	46 (26.4)	22 (26.1)	24 (26.6)	
Infection				0.113
Yes (%)	60 (34.5)	24 (28.6)	36 (40.0)	
No (%)	114 (65.5)	60 (71.4)	54 (60.0)	
Initial Blood Glucose mmol mean (SEM)	11.7 (6.1)	9.9 (4.6)	13.4 (6.9)	<0.001*
Mean Blood Glucose mmol (SEM)	10.1 (1.3)	9.0 (0.73)	11.9 (0.93)	<0.001*
Max Blood Glucose mmol mean (SEM)	15.6 (3.2)	13.8 (2.4)	17.5 (2.8)	<0.001*
Pre-operative HbA1c mmol/mol (n = 171)	8.4 (2.0)	7.7 (1.6)	9.2 (2.0)	<0.001*
Mortality				0.218
Yes	6 (3.4%)	4 (4.7%)	2 (2.2%)	
No	168 (96.6%)	80 (95.3%)	88 (97.8%)	

Of the whole cohort there were 92 individually reported infections with 10 patients having multiple infections. Types of infection reported were: deep and superficial surgical site infections (SSI), respiratory, urinary tract, systemic and other infections. Sixty (34%) patients had at least one documented infection in the six-week post-operative period. There was no statistically significant difference in the number of patients who developed an infection in the optimal peri-operative control group (n = 24; 14%) compared to sub-optimal peri-operative control group (n = 36; 21%) (p = 0.113).

**Figure 1:**
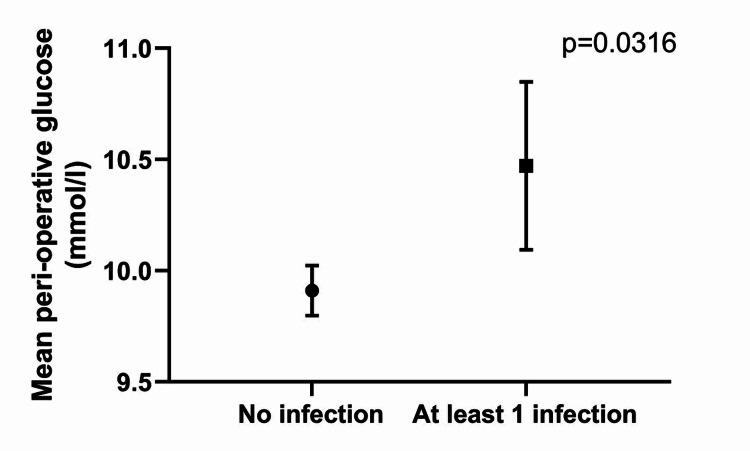
Mean perioperative blood glucose values and presence of post-operative infection

However, the value of the mean peri-operative glucose was higher in the patients who went on to develop at least one infection (mean glucose 10.4 mmol/l) compared to those that did not develop any infection (9.9 mmol/l), p = 0.0316 (Figure 1).

Regarding long-term pre-operative glycemic control we recorded HbA1c in 171 of the patients. Of these 130 (76%) were classified as having suboptimal control with HbA1c >7% and 41 (24%) were classified as having optimal long-term glucose control HbA1c ≤ 7% (Table 2). Of the 59 patients who developed post-operative infection 13 (31.7%) were in the optimal long-term control group, and 46 (35.3%) in the sub-optimal long-term control group, which was not statistically significant (p = 0.667).

**Table 2:** Long-term pre-operative blood glucose control according to HbA1c

Variable	All Patients, n = 171 (%)	Optimal control: HbA1c ≤7%, n = 41	Sub-optimal control: HbA1c >7%, n = 130	p-value
Infection				0.667
Yes (%)	59 (34.5)	13 (31.7)	46 (35.3)	
No (%)	112 (65.5)	28 (68.3)	84 (64.6)	
Mortality				0.304
Yes (%)	6 (3.4)	2 (4.5)	4 (3.0)	
No (%)	168 (96.6)	42 (95.5)	126 (97.0)	

**Figure 2:**
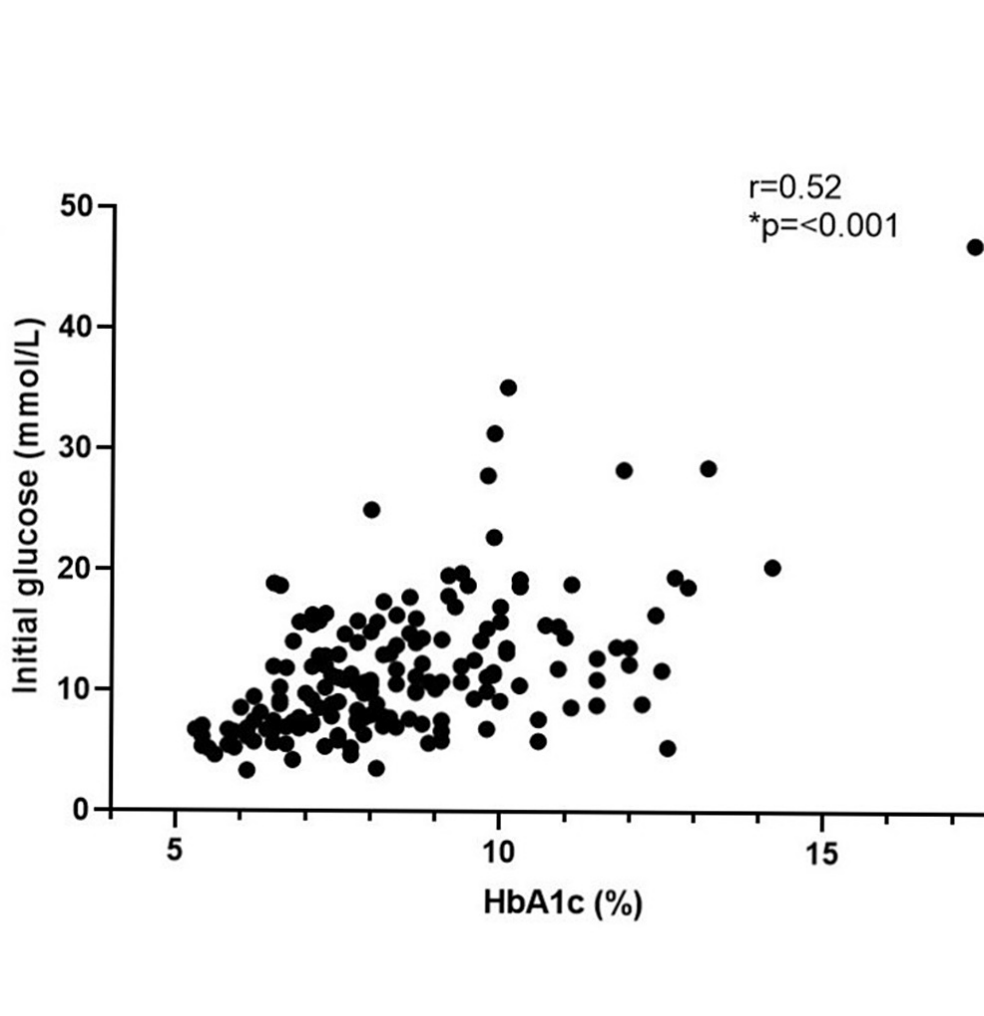
Scatter plot of correlation of HbA1c (n = 171) with mean initial glucose

**Figure 3:**
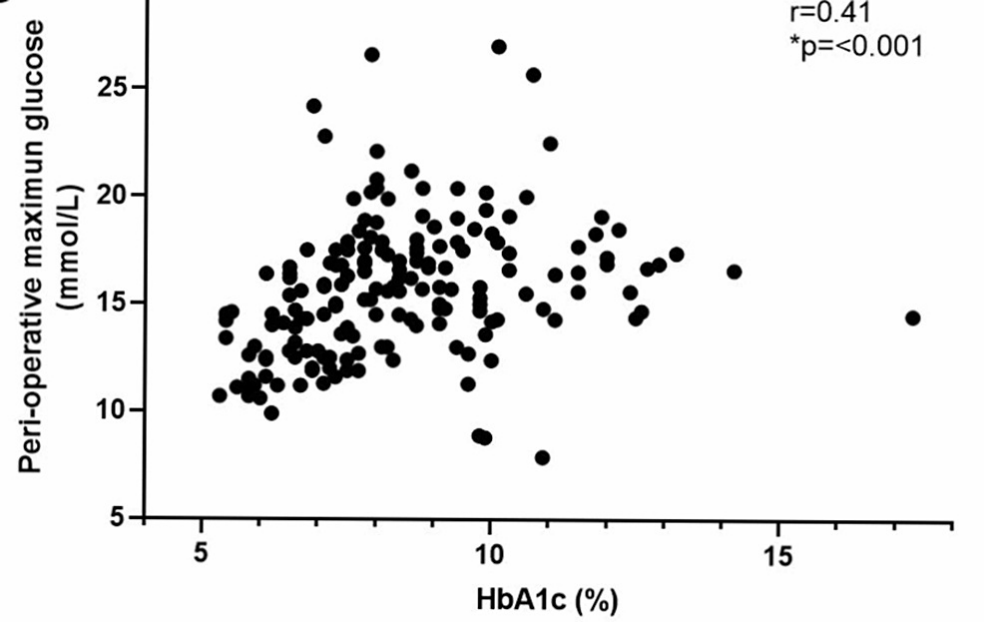
Scatter plot of correlation of HbA1c (n = 171) with mean maximum glucose

**Figure 4:**
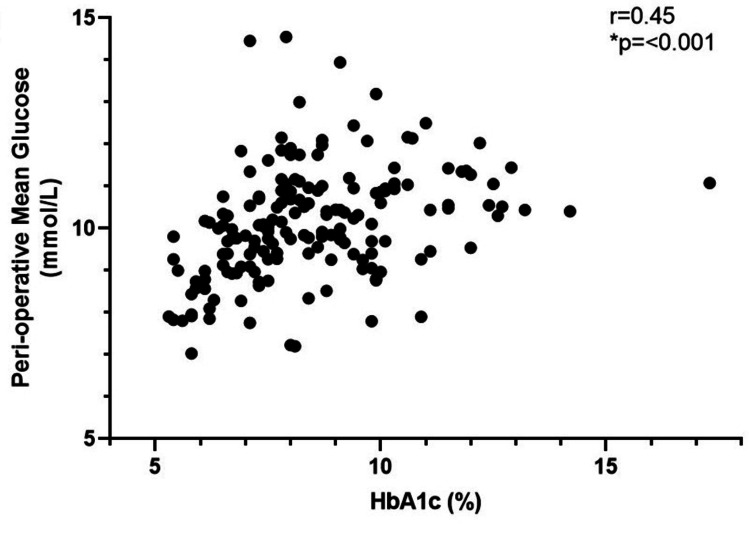
Scatter plot of correlation of HbA1c (n = 171) with mean peri-operative glucose

Looking at the influence of pre-operative glucose control on the ability to control perioperative glucose, we correlated individual HbA1c values with the mean initial glucose (Figure 2), mean maximum glucose (Figure 3), and the mean peri-operative glucose (Figure 4). There was a positive correlation with initial glucose (r = 0.52, p=<0.001), maximum recorded glucose (r = 0.41, p=<0.001) and mean peri-operative glucose (r = 0.45, p=<0.001).

## Discussion

In a review of over 30 million patients having surgery, diabetes ranks as one of the high-risk factors for post-operative infection [8]. Berghe et al. focused on tight glycemic control (4.4 to 6.1 mmol) and reduced major infections post-operatively by 50% [9]. Such tight control was not adopted by all centres. Castigliano et al. showed less complication and less infections with moderate glucose control of 6.7 to 9.7 mmol [10]. A study by Zerr et al. showed that the prolonged (48 hours post-operatively) use of intravenous insulin allows better glycemic control and improves infection outcomes post cardiac surgery [11].

In our study, we found that when dividing the patients into two groups based on their perioperative glycemic control, the data showed no statistical significance with postoperative infection. However, the mean peri-operative glucose was lower in those who did not develop post-operative infection. We believe that tight glycemic control is an important factor but having a mean cut-off value of 10 mmol for our groups was too high. If we had set a lower mean glucose we would have shown less infection with tighter control as shown in Figure 1.

Studies have looked at pre-operative HbA1c and the effect on infection. One study of 2,200 patients having non-urgent surgery compared HbA1c above and below 6.5% and showed no difference in post-operative infection [5]. Faritous et al. compared HbA1c above and below 7% for 216 cardiac surgery patients and showed fewer infections in the optimal HbA1c group [12]. One of our secondary objectives compared HbA1c above and below 7% but did not see any difference in the number of patients developing infection compared with the optimal long-term blood glucose control group as demonstrated by an HbA1c below 7%. However, we did see clearly that patients with higher HbA1c were more likely to develop post-operative infection (Table 1). So again, either our cut-off value for HbA1c of 7% was too high or the group with optimal long-term glucose control (n = 41, 24%) was too small.

Our other secondary objective looked at the link between HbA1c and the ability to achieve optimal peri-operative glycemic control. A review by Goh specifically looked at factors causing mediastinitis and showed that achieving good glycemic control was a significant factor [13]. A longitudinal study of 21,000 cardiac patients was able to demonstrate that in the period of stricter glycemic control the incidence of deep sternal wound infection fell from 3.0% to 1.0% [14]. Data from our own institution had shown the incidence of only SSI was no different when assessed by long-term glycemic control as measured by HbA1c [15]. In this study we have shown that a lower HbA1c before surgery was strongly correlated with improved peri-operative glucose control (Table 1). Therefore, controlling pre-operative HbA1c levels (long-term glycemic control) could be a key factor in achieving optimal glycemic control in the perioperative period.

There were limitations in this study. First, our peri-operative mean glucose value cut-off point of 10 mmol glucose was probably too high as the patients who remained infection free were found to have a mean glucose <10 mmol/l. Second was that in our sample only a small number of diabetic patients had optimal long-term control group. Also, the high incidence of infection overall suggests other factors than glycemic control were responsible (such as blood transfusion, antibiotic policy) and therefore infection was prevalent in all our groups. One of the strengths of the study was the fact that all cardiac surgical patients in the allocated period were assessed for diabetes and included in the study.

## Conclusions

The majority of our diabetic patients presented for cardiac surgery with sub-optimal long-term glucose control. This combined with the stress hyperglycaemia associated with cardiac surgery resulted in less than 50% of our patients achieving optimal peri-operative glycemic control. However, we were not able to find a correlation between our optimal and sub-optimal groups and post-operative infection. Importantly, this study did show that patients with higher HbA1c had more infections and that better peri-operative glucose was seen in patients with lower HbA1c which supports the need for efforts to improve long-term glucose control prior to undergoing cardiac surgery and should be part of the planning if time allows. In view of the high incidence of diabetes in Saudi Arabia this work demonstrates the importance of good pre- and peri-operative glucose control in minimizing post-operative infection.
